# Root-Zone CO_2_ Concentration Affects Partitioning and Assimilation of Carbon in Oriental Melon Seedlings

**DOI:** 10.3390/ijms231810694

**Published:** 2022-09-14

**Authors:** Xintong Han, Yuna Jing, Chuanqiang Xu, Lijia Gao, Minghui Li, Yiling Liu, Hongyan Qi

**Affiliations:** 1 College of Horticulture, Shenyang Agricultural University, Shenyang 110866, China; 2 Key Laboratory of Protected Horticulture Ministry of Education, Shenyang 110866, China; 3 National & Local Joint Engineering Research Center of Northern Horticultural Facilities Design & Application Technology, Shenyang 110866, China

**Keywords:** root-zone CO_2_, oriental melon, ^13^C stable isotope tracing, carbon assimilation

## Abstract

Root-zone CO_2_ is essential for plant growth and metabolism. However, the partitioning and assimilation processes of CO_2_ absorbed by roots remain unclear in various parts of the oriental melon. We investigated the time at which root-zone CO_2_ enters the oriental melon root system, and its distribution in different parts of the plant, using ^13^C stable isotopic tracer experiments, as well as the effects of high root-zone CO_2_ on leaf carbon assimilation-related enzyme activities and gene expressions under 0.2%, 0.5% and 1% root-zone CO_2_ concentrations. The results showed that oriental melon roots could absorb CO_2_ and transport it quickly to the stems and leaves. The distribution of ^13^C in roots, stems and leaves increased with an increase in the labeled root-zone CO_2_ concentration, and the δ^13^C values in roots, stems and leaves increased initially, and then decreased with an increase in feeding time, reaching a peak at 24 h after ^13^C isotope labeling. The total accumulation of ^13^C in plants under the 0.5% and 1% ^13^CO_2_ concentrations was lower than that in the 0.2% ^13^CO_2_ treatment. However, the distributional proportion of ^13^C in leaves under 0.5% and 1% ^13^CO_2_ was significantly higher than that under the 0.2% CO_2_ concentration. Photosynthetic carbon assimilation-related enzyme activities and gene expressions in the leaves of oriental melon seedlings were inhibited after 9 days of high root-zone CO_2_ treatment. According to these results, oriental melon plants’ carbon distribution was affected by long-term high root-zone CO_2_, and reduced the carbon assimilation ability of the leaves. These findings provide a basis for the further quantification of the contribution of root-zone CO_2_ to plant communities in natural field conditions.

## 1. Introduction

In agricultural production, improper irrigation, root respiration, microbial activities and the decomposition of various types of organic matter in the soil will lead to the enrichment of root-zone CO_2_ and a decrease in O_2_ content. This greatly impacts the growth and development of plants [1,2,3,4,5,6]. The rhizosphere has unique physicochemical and biological properties, which can regulate water absorption and nutrients and affect the reproduction of microorganisms [7,8,9]. Previous studies have shown that CO_2_ can be absorbed and fixed by roots, dissolved in the soil to form inorganic carbon, and then transported to stems and leaves to participate in photosynthesis and promote an increase in the total carbon content of plants [10,11,12,13]. The source and destination of carbon in plants and the transport speed of carbon assimilates can be detected by ^13^CO_2_ stable isotope tracing technology [14,15,16,17]. Studies have shown that absorbed CO_2_ in shoots can be quickly transported in plants, but since it takes time for them to transport photosynthates from the stems to the roots, more time is required to allocate ^13^C to the roots than to the stems and leaves [18,19]. CO_2_ is transported from the roots to the stems and leaves, where ^13^CO_2_ flows out of the leaf surface and diffuses into the atmosphere [20,21]. The ^13^C tracer of Camptotheca acuminate seedlings showed that the soluble inorganic carbon absorbed by roots could be used as a carbon source for photosynthesis, affecting the formation of photosynthates [22].

Carbon assimilation is an enzymatic reaction that involves a variety of enzymes. Ribulose-1,5-bisphosphate carboxylase/oxygenase (Rubisco) is the key enzyme in photosynthesis, and the main limiting factor regarding photosynthetic CO_2_ assimilation in C_3_ plants. Many factors affect its activity [23,24]. Rubisco activating enzyme (RCA) activity can determine the Rubisco carboxylation efficiency and limit plant photosynthesis [25]. Transketolase (TK) was found to be involved in photosynthetic carbon fixation, and its activity significantly affects the photosynthetic rate [26]. Fructose-1,6-diphosphate esterase (FBPase) is an essential enzyme whose activity directly impacts carbohydrate accumulation and photosynthetic efficiency [27]. The regeneration of ribulose-1,5-diphosphate (RuBP) caused by sedoheptulose-1,7-bisphosphatase (SBPase) regulates the inflow of carbon [28]. Phosphoglycerate kinase (PGK) is highly conserved, and is involved in glycolysis and photosynthesis during photosynthetic carbon fixation [29]. Phosphoribulokinase (PRK) is a vital enzyme in the Calvin cycle that is involved in photosynthesis [30]. The gene expressions of carbon assimilation-related enzymes will affect related enzyme activities, thus affecting photosynthesis. Studies have shown that transgenic modified tobacco plants overexpress photosynthetic carbon assimilated FBPase, SBPase and inorganic carbon transporter B (ict B), and photosynthesis in these plants was significantly enhanced [31].

The oriental melon (*Cucumis melo* var. *makuwa* Makino) is very sensitive to rhizosphere gas. Previous studies have found that rhizosphere gas often affects plant growth and fruit quality in facility cultivation [32,33]. The present study aimed to reveal the distribution of carbon absorbed by roots in plants, and the changes in enzyme activities and gene expression related to carbon assimilation under the conditions of elevated carbon dioxide in the root zone of oriental melon seedlings. We utilized ^13^C stable isotope labeling technology in order to explore the effects of high root-zone CO_2_ on the carbon absorption and carbon assimilation in oriental melon. The study provides a theoretical reference for further investigations into the response mechanisms of oriental melon root to high root-zone CO_2_ and the regulation of the rhizosphere gas environment.

## 2. Results

### 2.1. Root-Zone ^13^CO_2_ Concentration Affects the Abundance of ^13^C in Different Positions of Oriental Melon Plants

**Figure 1:**
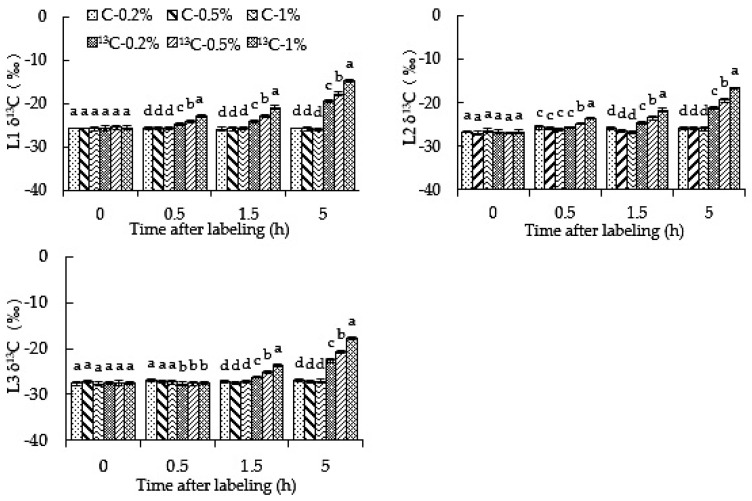
High root-zone ^13^CO_2_ affects the abundance of ^13^C in roots and different aboveground nodes of oriental melon plants. L1 represents the roots, L2 represents the first and second real-leaf and the first and second stems of the plant, and L3 represents other leaves and stems. Standard errors (SEs) of the means are represented by error bars. Different letters in the same row indicate significance at the 0.05 level among treatments.

The δ^13^C of labeled (^13^C-0.2%,^13^C-0.5% and ^13^C-1%) treatments at L1, L2 and L3 increased with the extension of feeding time (Figure 1). The δ^13^C of L1 with labeled treatments was significantly higher than that of unlabeled (C-0.2%, C-0.5% and C-1%) treatments after 0.5 h. This can be explained by the fact that ^13^C was detected in L1 of the labeled treatment. In L2, the δ^13^C of ^13^C-0.5% and ^13^C-1% treatments were significantly higher than those of unlabeled treatments at 0.5 h, i.e., ^13^C was detected, while the δ^13^C of the ^13^C-0.2% treatment was significantly higher than that of unlabeled treatments at 1.5 h, and the detection time of ^13^C was later than that of ^13^C-0.5% and ^13^C-1% treatments. In L1 and L2, the δ^13^C of ^13^C-0.5% and ^13^C-1% labeled treatments were significantly higher than that of the ^13^C-0.2% labeled treatment. ^13^C-1% labeled was significantly higher than that of ^13^C-0.5% during the labeling period, and the differences were enhanced with the extension of the feeding time. This shows that the greater the label concentration, the faster the transportation from root to shoot. At the L3 site, 1.5 h after feeding, the δ^13^C value of labeled treatments was significantly higher than that of unlabeled treatments, and the ^13^C-0.5% and ^13^C-1% treatments were significantly higher than that of ^13^C-0.2%, while ^13^C-1% treatment was significantly higher than that of ^13^C-0.5%. The results showed that oriental melon roots could absorb CO_2_ and rapidly transport it upward; moreover, the higher the root-zone CO_2_ concentration, the more CO_2_ is absorbed by roots, and the faster the transportation speed to the aboveground region.

### 2.2. Root-Zone ^13^CO_2_ Concentration Affects the Abundance of ^13^C in Roots, Stems and Leaves of Oriental Melon

**Figure 2:**
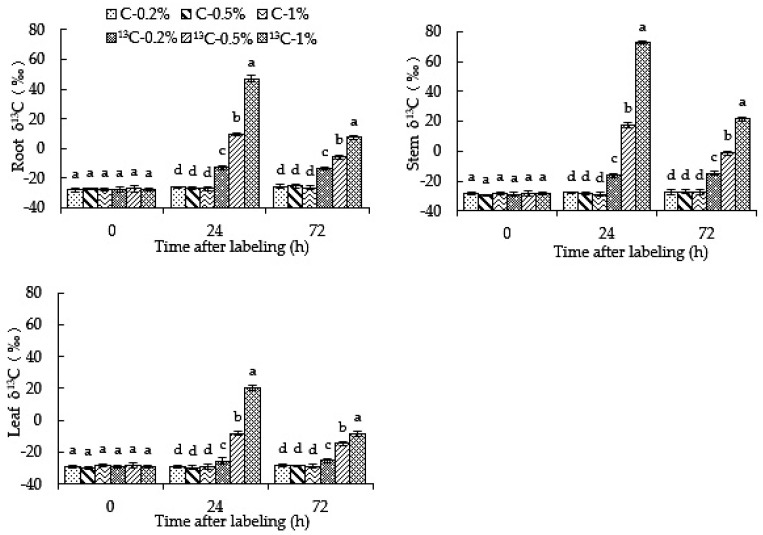
High root-zone ^13^CO_2_ affects the ^13^C abundance in roots, stems and leaves of oriental melon. Standard errors (SEs) of the means are represented by error bars. Different letters in the same row indicate significance at the 0.05 level among treatments.

It can be seen from Figure 2 that at 24 h and 72 h after labeling, the δ^13^C values of the ^13^C-0.2%,^13^C-0.5% and ^13^C-1% treatments in roots, stems and leaves were significantly higher than those of unlabeled treatments (C-0.2%, C-0.5% and C-1%), and that there was no significant difference between unlabeled treatments; this indicates that ^13^C of labeled treatments in root-zone ^13^CO_2_ could be detected in roots, stems and leaves. The δ^13^C value in roots, stems and leaves showed an initial increase, followed by a decrease with the extension of the labeling time, and reached a peak at 24 h. The δ^13^C values in roots, stems and leaves were significantly higher in the ^13^C-0.5% and ^13^C-1% treatments than that in the ^13^C-0.2% treatment; moreover, the ^13^C-1% treatment was significantly higher than that of ^13^C-0.5% at 24 h and 72 h, and the difference decreased with an increase in labeling time. The results showed that the root-zone CO_2_ concentration and treatment time could affect the enrichment degree of root, stem and leaf to new carbon absorbed by the roots of oriental melon plants.

### 2.3. Root-Zone ^13^CO_2_ Concentration Affects the Distribution of Carbon in Roots, Stems and Leaves of Oriental Melon

**Figure 3:**
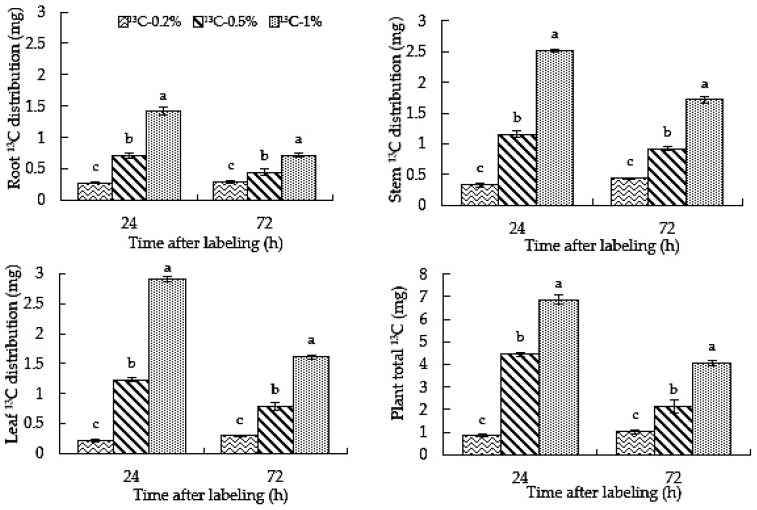
High root-zone ^13^CO_2_ affects the distribution of C in roots, stems and leaves of oriental melons. Standard errors (SEs) of the means are represented by error bars. Different letters in the same row indicate significance at the 0.05 level among treatments.

As can be seen from Figure 3, with the extension of treatment time, the ^13^C-0.2% treatment increased the ^13^C distribution in roots, stems and leaves, and the total amount of ^13^C in plants. The ^13^C distribution and total amount of ^13^C-0.5% and ^13^C-1% treatments increased initially and then decreased. The ^13^C distributions of ^13^C-0.5% and ^13^C-1% treatments were significantly higher than that of the ^13^C-0.2% treatment at 24 h and 72 h. Moreover, the ^13^C distribution of ^13^C-1% in roots, stems and leaves was significantly higher than that for ^13^C-0.5%. The results showed that in ^13^C-0.5% and ^13^C-1% labeled treatments, roots absorbed more carbon than in the ^13^C-0.2% treatment, and the ^13^C allocation increased before decreasing with the extension of the treatment time. The root-zone CO_2_ concentration and treatment time affected the carbon allocation of each part of the plant.

### 2.4. Root-Zone ^13^CO_2_ Concentration Affects the Distribution Proportion of Carbon in Roots, Stems and Leaves of Oriental Melon

**Figure 4:**
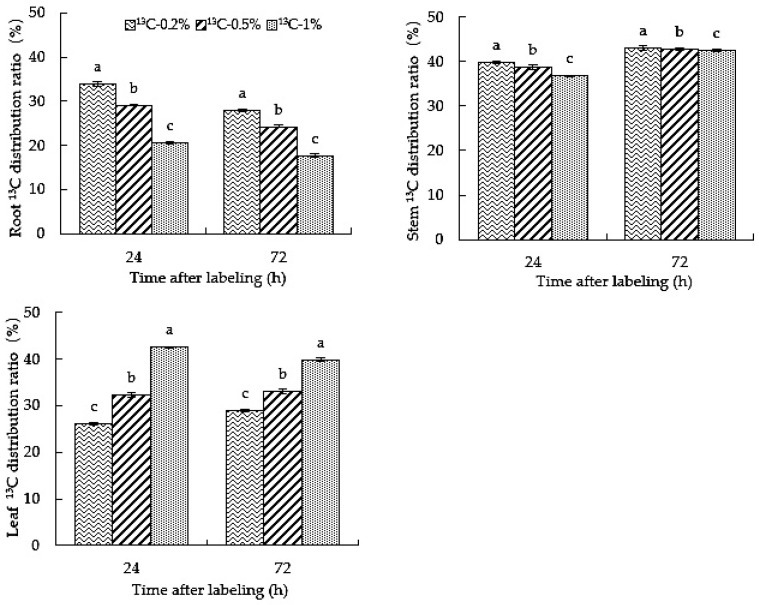
High root-zone ^13^CO_2_ affects the distribution proportion of carbon in roots, stems and leaves. Standard errors (SEs) of the means are represented by error bars. Different letters in the same row indicate significance at the 0.05 level among treatments.

The distribution ratio of labeled ^13^C in each part of the plant can indicate the distribution of carbon absorbed by the roots. It can be seen from Figure 4 that at 24 h and 72 h after labeling, the ^13^C distribution proportion of ^13^C-0.2% was significantly higher than that of the ^13^C-0.5% and ^13^C-1% treatments in stems and roots. Nevertheless, the distribution ratios of ^13^C in ^13^C-0.5% and ^13^C-1% treatments were higher than that of ^13^C-0.2% in leaves, and ^13^C-1% was significantly higher than ^13^C-0.5%. At 72 h after labeling, the distribution ratios of ^13^C-0.2%, ^13^C-0.5% and ^13^C-1% treatments were the highest in the stems and the lowest in the roots. With the extension of the feeding time, the distributional proportion of ^13^C in ^13^C-0.2%, ^13^C-0.5% and ^13^C-1% treatments decreased in roots but increased in stems; the ^13^C-0.2% treatment in leaves showed an increasing trend, while it showed a decreasing trend under ^13^C-1% treatment. The results showed that the distribution of CO_2_ absorbed by roots in different parts of oriental melon was affected differently by the root-zone CO_2_ concentration and treatment time: the higher the root-zone CO_2_ concentration, the more significant the proportion of carbon distribution in aboveground leaves.

### 2.5. Root-Zone ^13^CO_2_ Concentration Affects the Accumulation of Biomass in Roots, Stems and Leaves of Oriental Melon

**Figure 5:**
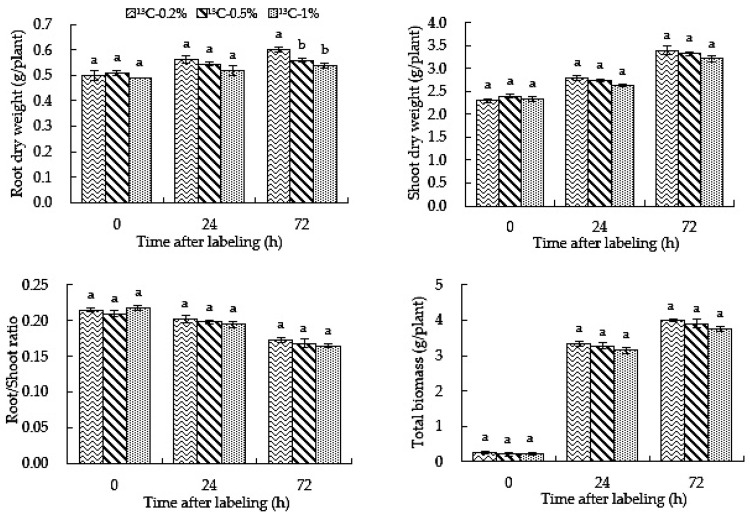
High root-zone ^13^CO_2_ affects the accumulation of biomass in roots and shoots of oriental melon. Standard errors (SEs) of the means are represented by error bars. Different letters in the same row indicate significance at the 0.05 level among treatments.

The results showed that the dry mass of roots, stems and leaves increased with an increase in treatment time, as shown in Figure 5. At 24 h after labeling, the difference in the dry mass of roots between the three CO_2_ concentration labeling treatments was not significant; however, at 72 h after labeling, the ^13^C-0.5% and ^13^C-1% treatments yielded significantly lower values than the ^13^C-0.2% treatment. During the treatment period, there was no significant difference in the dry weight of shoots, root/shoot ratio and total biomass between different concentration treatments. The results showed that an elevated root-zone CO_2_ concentration inhibited the accumulation of dry matter in roots with an increase in treatment time, but the effect on other types of biomass accumulation was insignificant.

### 2.6. Root-Zone ^13^CO_2_ Concentration Affects the Total Carbon Content in Roots, Stems and Leaves of Oriental Melon

**Figure 6:**
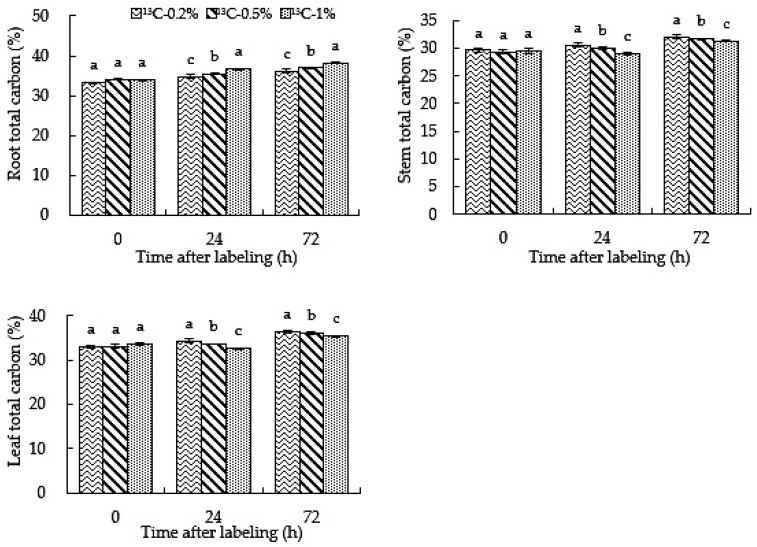
High root-zone ^13^CO_2_ affects the total carbon content in roots, stems and leaves of oriental melon. Standard errors (SEs) of the means are represented by error bars. Different letters in the same row indicate significance at the 0.05 level among treatments.

Carbon content can indicate the ability of plants to fix and store carbon. It can be seen from Figure 6 that, with an increase in treatment time, the carbon content in the roots and stems under the ^13^C-0.5%, ^13^C-1% and ^13^C-0.2% treatments increased. During the treatment, the carbon content under the ^13^C-0.2% treatment was significantly lower than those under ^13^C-0.5% and ^13^C-1% treatments in roots, and the ^13^C-1% treatment yielded significantly higher values than ^13^C-0.5%. The ^13^C-0.2% treatment yielded significantly higher values than ^13^C-0.5% and ^13^C-1% treatments in stems and leaves. The results showed that the higher the root-zone CO_2_ concentration of oriental melon, the more carbon became fixed in the root system.

### 2.7. Root-Zone ^13^CO_2_ Concentration Affects the Accumulation of Carbon in Roots, Stems and Leaves of Oriental Melon

**Figure 7:**
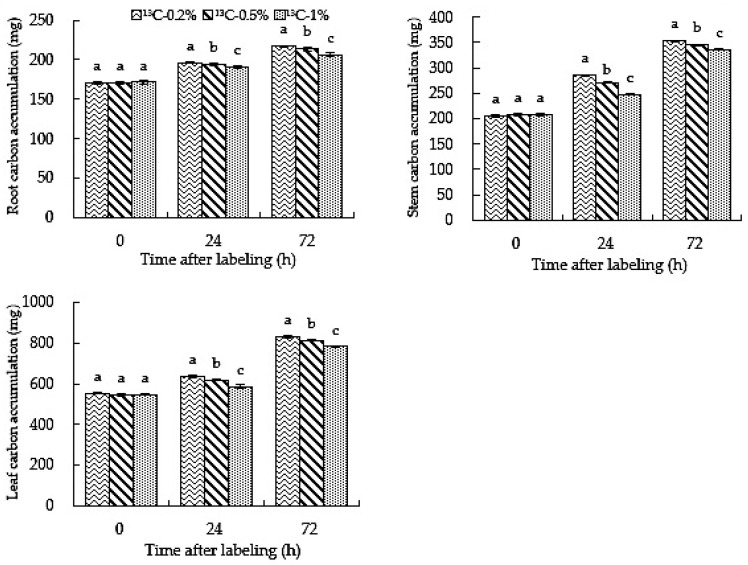
High root-zone ^13^CO_2_ affects carbon accumulation in roots, stems and leaves of oriental melon. Standard errors (SEs) of the means are represented by error bars. Different letters in the same row indicate significance at the 0.05 level among treatments.

The amount of carbon accumulation in the plant is the most intuitive indicator of carbon fixation. It can be seen from Figure 7 that the carbon accumulation in roots, stems and leaves increased with the increase in treatment time. The carbon accumulation under the ^13^C-0.2%, ^13^C-0.5% and ^13^C-1% treatments was in the order of leaf > stem > root. The carbon accumulation was affected by the carbon content and dry matter quality. The carbon content and dry matter accumulation of various organs at different concentrations increased with treatment time. Therefore, with the growth of oriental melon, the carbon accumulation of roots, stems and leaves treated with ^13^C-0.2%,^13^C-0.5% and ^13^C-1% increased. The carbon accumulation in roots, stems and leaves under ^13^C-0.2% treatment was significantly higher than those of the ^13^C-0.5% and ^13^C-1% treatments, and ^13^C-0.5% treatment yielded significantly higher values than ^13^C-1%. In conclusion, high root-zone CO_2_ inhibited the carbon accumulation of oriental melon roots, stems and leaves. In other words, high root-zone CO_2_ inhibited the carbon fixation in oriental melon, thus affecting carbon assimilation; the higher the root-zone CO_2_ concentration, the more significant the inhibitory effect.

### 2.8. Root-Zone CO_2_ Concentration Affects the Activities of Carbon Assimilation-Related Enzymes in Oriental Melon

**Figure 8:**
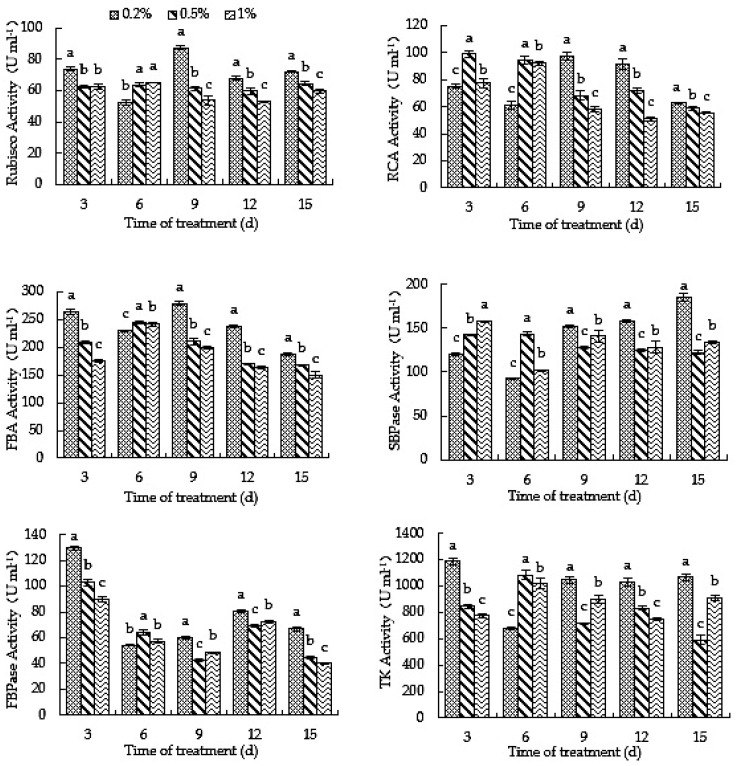
High root-zone CO_2_ affects the activities of carbon assimilation-related enzymes in oriental melon. Standard errors (SEs) of the means are represented by error bars. Different letters in the same row indicate significance at the 0.05 level among treatments.

Rubisco, RCA, TK, FBA, SBPase, FBPase and other enzymes in plants are mainly involved in the dark reaction of photosynthesis, and play a role in carbon assimilation. As shown in Figure 8, on the third day of treatment, the activity of Rubisco, FBA, FBPase and TK in the 0.5% and 1% root-zone CO_2_ treatments was significantly lower than that in the 0.2% treatment, and while the activity of SBPase was significantly higher than that in the 0.2% treatment, the RCA activity in the 0.5% treatment was significantly higher than that in the 0.2% treatment. On the sixth day of treatment, the activity of Rubisco, RCA, FBA, SBPase and TK in the 0.5% and 1% treatments was significantly higher than that in the 0.2% treatment, and FBPase activity in the 0.5% treatment was significantly higher than that in 0.2%. After the ninth day of treatment, the activity of Rubisco, RCA, FBA, SBPase, FBPase and TK in the 0.5% and 1% treatments was significantly lower than that in the 0.2% treatment. The results showed that a high root-zone CO_2_ concentration could significantly affect the activity of carbon assimilation-related enzymes in oriental melon, and 0.5% and 1% root-zone CO_2_ concentrations could significantly inhibit the activity of carbon assimilation-related enzymes after 9 days: the higher the CO_2_ concentration, the more significant the inhibitory effect, thus affecting the carbon assimilation of oriental melon seedlings. This is also one of the reasons that high root-zone CO_2_ treatment inhibited the photosynthesis of oriental melon seedlings.

### 2.9. Root-Zone CO_2_ Concentration Affects the Expression of Carbon Assimilation-Related Enzyme Genes in Oriental Melon

**Figure 9:**
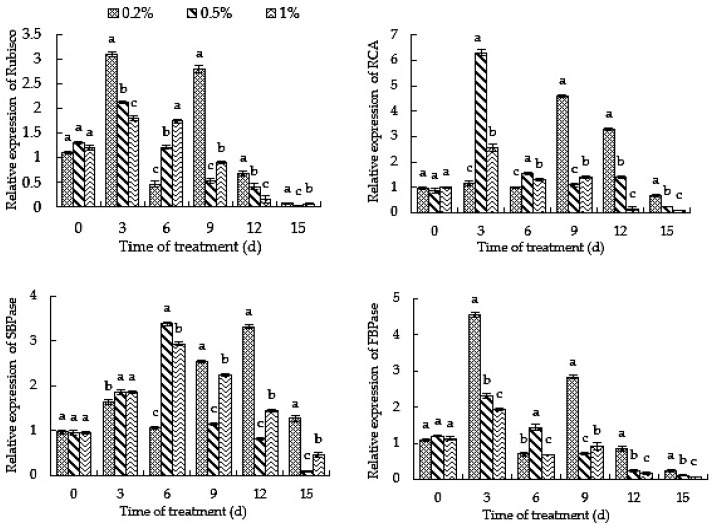

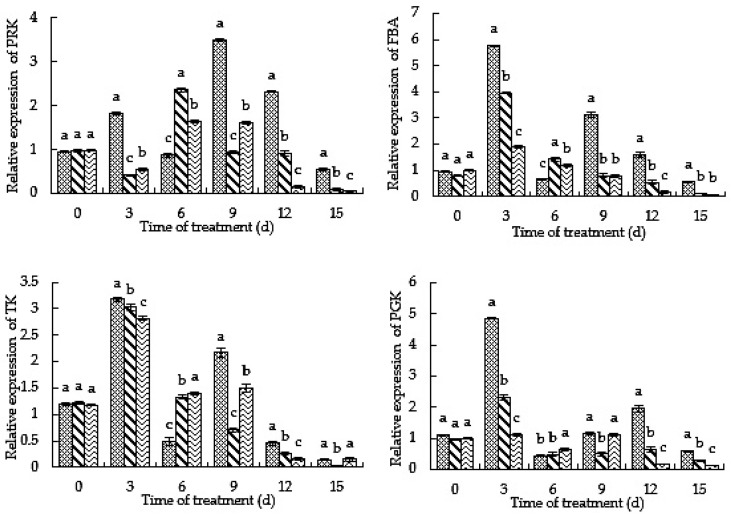
High root-zone CO_2_ affects the expressions of carbon assimilation-related enzyme genes in oriental melon. Standard errors (SEs) of the means are represented by error bars. Different letters in the same row indicate significance at the 0.05 level among treatments.

Plant photosynthetic carbon assimilation is directly affected by the activities of various carbon assimilation-related enzymes. At the same time, the activities of carbon assimilation-related enzymes are also affected by the gene expressions of carbon assimilation-related enzymes in plants. Figure 9 shows the relative expressions of carbon assimilation-related enzyme genes in oriental melon seedlings under high root-zone CO_2_ treatment. It can be seen from the figure that high root-zone CO_2_ had a significant effect on the expressions of carbon assimilation-related enzyme genes in the leaves of oriental melon seedlings. The expression levels of *Cm*RCA, which determines the carboxylation efficiency of Rubisco, and *Cm*SBPase, which regulates carbon influx, were significantly higher in the 0.5% and 1% treatments than in the 0.2% treatment at 3–6 days, and significantly lower in the 0.5% and 1% treatments than the 0.2% treatment after 9 days. This indicates that a high root-zone CO_2_ concentration can promote *Cm*RCA and *Cm*SBPase expressions in the short term, and increase the syntheses of SBPase and RCA; the Rubisco carboxylation efficiency and carbon inflow also increased. Long-term high root-zone CO_2_ concentration treatment can inhibit gene expression, negatively regulating the syntheses of SBPase and RCA. The expressions of *Cm*Rubisco, *Cm*PRK and *Cm*FBA, which regulate photosynthesis and *Cm*FBPase, which affects carbohydrate accumulation and photosynthetic efficiency, were significantly lower in the 0.5% and 1% treatments on the third day than in the 0.2% treatment, significantly higher than in the 0.2% treatment on the sixth day and significantly lower on the ninth day than in the 0.2% treatment. The expression of *Cm*FBPase was only observed under the 0.5% treatment, which was significantly higher than 0.2% on the sixth day. The expression of *Cm*TK, involved in photosynthetic carbon fixation, under the 0.5% and 1% treatments was significantly lower than that of the 0.2% treatment on the third day, significantly higher than that of the 0.2% treatment on the sixth day and significantly lower than that of the 0.2% treatment on the ninth to the twelfth day. The expression of *Cm*PGK in the 0.5% and 1% treatments on the third day was significantly lower than that in the 0.2% treatment, and that in the 1% treatment on the sixth day was significantly higher than that in the 0.2% treatment; *Cm*PGK was significantly lower in the 0.5% and 1% treatments than in the 0.2% treatment after 12 days, which indicated that long-term high root-zone CO_2_ treatment could cause the expressions of *Cm*Rubisco, *Cm*FBPase, *Cm*FBA, *Cm*PRK, *Cm*TK and *Cm*PGK to be down-regulated, thus inhibiting the syntheses of related enzymes and affecting the carbohydrate accumulation and photosynthetic rate. In conclusion, after 9 days of high root-zone CO_2_ concentration treatment, the expressions of carbon assimilation-related enzyme genes in oriental melon were down-regulated, and the activities of carbon assimilation-related enzymes were inhibited, thus inhibiting carbon assimilation in oriental melon.

## 3. Discussion

### 3.1. High Root-Zone CO_2_ Affects Oriental Melon’s Root Carbon Absorption and Distribution

The ^13^C stable isotope is sensitive, simple and can be easily located and quantified. It has become an important means to study carbon’s absorption, distribution and transformation. The use of ^13^C stable isotope labeling technology is beneficial not only in the study of the effects of high root-zone CO_2_ on plants, but also in tracking the absorption of rhizosphere carbon by roots. The results showed that the root system of oriental melon could absorb CO_2_ and transport it upward rapidly. Moreover, ^13^C could be detected in all parts after treatment, and δ^13^C increased with treatment time, but the detection time of each part was different. This may be because the upward transport of CO_2_ can only be completed after it is absorbed by the root system for a certain time; previous studies estimated that 65–99% ^13^C was released to the atmosphere in 9 h to 4 weeks [34]. In this test, at the same time and in the same part, the δ^13^C in the 0.5% and 1% root-zone ^13^CO_2_ treatments is significantly higher than that in the 0.2% treatment, ^13^C can be detected first in the 0.5% and 1% ^13^CO_2_ treatments, and the 1% treatment yields significantly higher values than 0.5%. The study found that higher dissolved carbon can be released under a high concentration of CO_2_ [35]. The assimilation of xylem-transported CO_2_ is affected by the CO_2_ concentration in the xylem: the higher the CO_2_ concentration, the greater the ^13^C enrichment and assimilation [36]. L2 treated with 0.5% and 1% root-zone ^13^CO_2_ showed ^13^C before 0.2% treatment, which may be due to more ^13^C being absorbed by roots treated with higher root-zone ^13^CO_2_ and the faster upward transport speed.

After ^13^CO_2_ labeling in the rhizosphere, ^13^C can be detected in all organs of oriental melon (Figure 2). This study showed that when the 0.2%, 0.5% and 1% root-zone ^13^CO_2_ labeling treatments reached 24 h, δ^13^C in roots increased, and roots could absorb CO_2_. The absorbed CO_2_ is transported upward to the stems and leaves as the substrate of photosynthesis, or is diffused directly from the leaves to the atmosphere, resulting in a reduction in ^13^C in the roots [22] and decreasing the δ^13^C value in the roots after 24~72 h. Long-term high root-zone CO_2_ treatment increases the concentration of CO_2_ in root cells [37], which may reduce the ability of roots to absorb ^13^CO_2_. Compared with 0.2% root-zone ^13^CO_2_ treatment, ^13^C in the 0.5% and 1% treatments was absorbed by roots and transported upward, to a greater extent under the 1% treatment than the 0.5%. Therefore, the ^13^C content in roots decreased more significantly, and the amount of ^13^C transported to stems and leaves decreased. High root-zone CO_2_ treatment will affect the transportation capacity of xylem to water and nutrient elements, slow down the transportation of ^13^C absorbed by roots to stems and leaves, release ^13^C via respiration in stems and leaves and increase the dry mass of stems and leaves; the result is that δ^13^C is diluted, leading to a decrease in δ^13^C in stems and leaves over 24~72 h. The increase in δ^13^C in stems and leaves in the 0.2% treatment may be due to the continuous upward transportation of ^13^C after being absorbed by the roots, and the upward transportation content is higher than its loss. During the labeling period of the 0.2% root-zone ^13^CO_2_ treatment, the size of δ^13^C was in the order of root > stem > leaf, which was consistent with the results of previous studies [38]. The δ^13^C value under the 0.5% and 1% ^13^CO_2_ treatments is in the order of stem > root > leaf, which may be due to the fact that the increase in root-zone ^13^CO_2_ concentration specifically promotes root growth in the short term [39]. Oriental melon seedlings have a higher transpiration rate, which facilitates the upward transport of carbon dioxide, enhances the photosynthesis of stems and intercepts the carbon dioxide diffused into the atmosphere [20].

In the process of treatment, the distribution of ^13^C in roots, stems and leaves showed that the 0.5% and 1% root-zone ^13^CO_2_ treatments were significantly more effective than the 0.2% treatment (Figure 3). With an increase in labeling time, the δ^13^C in stems and leaves under the 0.2% treatment increased, the δ^13^C in roots almost did not decrease within 24~72 h, and the dry mass in various organs increased. Therefore, the distribution of ^13^C in roots, stems and leaves increased. At 24~72 h, although the dry matter accumulation under the 0.5% and 1% treatments increased, the δ^13^C in each organ decreased, resulting in ^13^C distribution. Moreover, the 0.5% and 1% root-zone ^13^CO_2_ treatments significantly increased the ^13^C distribution ratio in leaves compared to the 0.2% treatment (Figure 4), which may be due to the fact that the CO_2_ content in the greenhouse could not completely fulfill the needs of oriental melon leaves for photosynthesis. The carbon that was absorbed in the rhizosphere was transported to the leaves as an alternate carbon source to participate in photosynthesis, or the high root-zone CO_2_ inhibited the photosynthesis in oriental melon [40,41]. The proportion of photosynthetic carbon allocated to the lower part of the ground during plant growth is reduced [42], which may cause more carbon to be absorbed by the root system and transported to the leaves for photosynthesis under high CO_2_ stress in the rhizosphere. Previous studies have shown that the distribution of ^13^C absorbed by plants is affected by many factors [43,44]. Both root-zone ^13^CO_2_ concentration and treatment time will affect carbon distribution.

Carbon content can be used to indicate the carbon fixation capacity of plants. Plants mainly absorb and assimilate a large amount of CO_2_ through photosynthesis. With an increase in treatment time, the carbon content in each organ increased. The carbon content of roots under the 0.5% and 1% treatments was significantly higher than that of the 0.2% treatment and lower than that of the 0.2% treatment in leaves. The reason for this may be that more carbon was absorbed by roots under the 0.5% and 1% root-zone CO_2_ treatments, resulting in an increase in carbon content in roots; alternately, it may have been an initial stress response or an increase in root-zone temperature, which is conducive to the transfer of photosynthetic products from leaves to roots [45]. The research shows that high root-zone CO_2_ treatment enhances root nitrogen metabolism, so it is necessary to provide a carbon source in the upper part and reduce the carbon content in leaves [46]. Therefore, the carbon content under the 0.5% and 1% root-zone CO_2_ treatments is higher than that under the 0.2% treatment in roots and lower than that under the 0.2% treatment in leaves.

The carbon accumulation in plants reflects the material accumulation from photosynthesis by plants using various growth factors [47]. Plant dry weight and carbon content determine the amount of carbon accumulation. In addition, photosynthetic carbon tends to accumulate in roots, stems and leaves during vegetative growth [48]. There was greater carbon content in roots treated with the 0.5% and 1% root-zone CO_2_ compared to those under the 0.2% treatment, and the dry matter accumulation was lower than that under the 0.2% treatment; meanwhile, the carbon content and dry matter accumulation of stems and leaves were lower than those under the 0.2% treatment; thus, 0.2% root-zone CO_2_ concentration treatment led to higher carbon accumulation in stems and leaves than for the 0.5% and 1% treatments. Although the carbon content of roots that were treated with 0.2% root-zone CO_2_ was lower than that of the 0.5% and 1% treatments, the dry matter accumulation was higher than that of the 0.5% and 1% treatments; thus, the carbon accumulation of roots treated with the 0.2% treatment was higher than that of the 0.5% and 1% treatments (Figure 7). The results showed that carbon accumulation was inhibited under a root-zone CO_2_ concentration greater than 0.5%, which inhibited carbon fixation and affected carbon assimilation. Studies have shown that with an increase in dissolved inorganic carbon in the rhizosphere of plants, biomass accumulation will increase, but the absorption of nutrients by plants may also change [49]. An increase in CO_2_ concentration significantly improves plants’ carbon absorption capacity and promotes plants’ carbon accumulation [50,51,52,53]. However, the carbon accumulation under high root-zone CO_2_ treatment was inhibited, which may have been due to the decline in plant photosynthetic capacity caused by high CO_2_ enrichment in the rhizosphere, which is not conducive to carbon accumulation in plant organs.

### 3.2. Elevated Root-Zone CO_2_ Affects Carbon Assimilation of Oriental Melon Seedlings

The Calvin cycle is the primary pathway of carbon assimilation in C_3_ plants. Rubisco, RCA, TK, FBPase, SBPase, FBA and other enzymes are the key enzymes in the Calvin cycle. Rubisco can be used to fix CO_2_ and determine the level of the net photosynthetic rate. It is the key enzyme in photosynthesis and the rate-limiting enzyme in CO_2_ assimilation [54]. RCA has little effect on the photosynthetic rate and can promote and stabilize Rubisco enzyme activity [25]. SBPase can maintain the regeneration of RuBP and the flow of carbon in the Calvin cycle, which plays an important role in carbon assimilation [28]. FBA controls the photosynthetic rate. A slight decrease in TK activity will significantly decrease the plant photosynthetic rate [26]. FBPase is a regulatory enzyme in the Calvin cycle and plays an important role in photosynthetic product transport [27]. Rubisco, RCA, FBPase and thioredoxin (Trx) affect plant photosynthesis [55]. Current studies have shown that root-zone CO_2_ enrichment can inhibit photosynthesis [56], but the internal mechanism of the effect of high root-zone CO_2_ on carbon assimilation-related enzymes remains to be studied. It was found that photosynthetic carbon assimilation enzyme activity would affect its carbon assimilation [57]. The activities of enzymes related to carbon assimilation affect the photosynthetic carbon assimilation of plants. This study found that, from the ninth day after treatment, the activity of Rubisco, RCA, TK, FBPase, SBPase and FBA decreased significantly, indicating that long-term 0.5% and 1% high root-zone CO_2_ treatments inhibited the activities of carbon assimilation-related enzymes, thus inhibiting photosynthesis in oriental melon seedlings. The gene expressions of carbon assimilation-related enzymes will affect enzyme activity, thus affecting the ability of plants to assimilate CO_2_ and then regulate carbon assimilation. After 9 days of treatment, the expressions of *Cm*RCA, *Cm*SBPase, *Cm*FBPase, *Cm*FBA, *Cm*PRK and *Cm*Rubisco under the 0.5% and 1% treatments were significantly lower than those in the 0.2% treatment, and the expressions of *Cm*TK and *Cm*PGK in the 0.5% and 1% treatments were significantly lower than those under 0.2% after 9–12 days and 12 days, respectively. The results showed that long-term high root-zone CO_2_ treatment decreased the expressions of essential enzyme genes in carbon assimilation, decreased the activities of carbon assimilation-related enzymes and inhibited photosynthetic carbon assimilation. Carbon assimilation is one of the main means of enrichment under the condition of elevated root-zone CO_2_ [58]. The above results reveal the effect of high root-zone CO_2_ on plant carbon assimilation from the perspective of carbon assimilation-related enzymes. In addition, the gene expressions of carbon assimilation-related enzymes are affected by high root-zone CO_2_, which regulates internal enzyme activities through gene expression, and then regulates the photosynthetic carbon assimilation of plants.

## 4. Materials and Methods

### 4.1. Plant Materials and Growth Conditions

Oriental melon, of the ‘Yumeiren’ cultivar, was grown aeroponically in the greenhouse at Shenyang Agricultural University, Shenyang, Liaoning, China. The test was carried out when the oriental melon seedlings grew to three real-leaf stages. Yamazaki nutrient solution for oriental melon was supplied through a pump. The nutrient solution was replaced every 4 days. When the seedlings grew to five real-leaf stages, oriental melon seedlings with uniform size were selected for isotope labeling. During the labeling period, the temperature was 25–30 °C in the daytime, with adequate illumination, and 15–20 °C at night; the relative humidity of the air was 50–60%.

### 4.2. Isotopic ^13^CO_2_ Feeding Experiment

**Figure 10:**
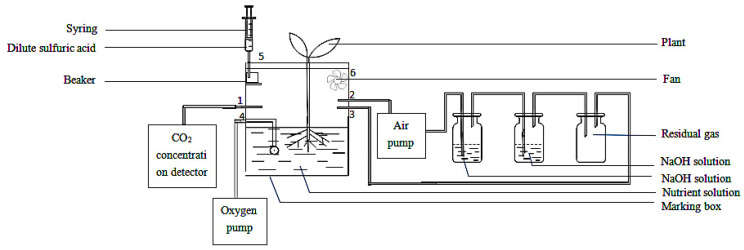
Isotope tracer processing system.

A ^13^C carbonic acid (Na_2_^13^CO_3_) stable isotopic tracer experiment was performed (as shown in Figure 10). The ^13^CO_2_ stable isotope marking box was composed of glass. The marking box’s length, width and height were 50 cm, 50 cm and 25 cm, respectively. A cultivation hole with a diameter of 2 cm was drilled every 10 cm above the marking box, and 9 seedlings could be planted in each marking box. A 100 mL beaker (containing labeled Na_2_^13^CO_3_) was fixed on the box’s inner wall. Na_2_^13^CO_3_ (99 atom% ^13^C) was used for feeding treatment, and Na_2_CO_3_ was used as the control for unlabeled CO_2_ treatment. Six holes with a diameter of 1 cm were set 5 cm above the side of the marking box, with a hole spacing of 10 cm. A rubber tube was installed in hole 1. One end of the rubber tube was sleeved with the sensor of a portable CO_2_ concentration detector (Keernuo Electronic Technology. Co., Ltd., Shenzhen, China, model GT-903), and the other end of the rubber tube was inserted into the middle of the liquid nutrient level and gas part to measure the ^13^CO_2_ (CO_2_) concentration in the marking box. The CO_2_ absorption device was connected through the rubber tube in hole 2. Before feeding, the gas in the feeding box was extracted through the rubber tube by the air pump, the CO_2_ component in the gas in the box was removed through the washing bottle containing NaOH solution, and then the other gases except CO_2_ were sent back to the feeding box through the rubber tube in hole 3. Hole 4 of the marking box was connected to the O_2_ increasing pump to maintain the O_2_ concentration of the root system in the marking box at a normal level. Hole 5 was connected to a syringe containing dilute sulfuric acid (2 mol L^−1^). When feeding began, dilute sulfuric acid was injected into the beaker containing Na_2_^13^CO_3_, and a particular concentration of ^13^CO_2_ (CO_2_) was produced after the reaction. A small fan was installed in hole 6 to ensure that the gas ^13^CO_2_ (CO_2_) concentration in the marking box was uniform. We sealed all interfaces of the marking box with sealant to keep the marking box closed during feeding. The outer layer of the marking box was covered with a black film during the treatment, and the plants were fixed on the cultivation hole with a rubber stopper to keep the root system within the marking box. We placed an appropriate amount of nutrient solution (pH 6.5~6.8) into the box to cause 1/3 of the root system of oriental melon to come into contact with the nutrient solution. The device obtained the national utility model patent (Patent No.: ZL 201920165969.2).

#### 4.2.1. Root-Zone CO_2_ Concentration Treatment

At the beginning of feeding, we used a syringe to inject 50 mL dilute sulfuric acid (2 mol L^−1^) into the beaker, which reacted with Na_2_^13^CO_3_(Na_2_^12^CO_3_) to produce ^13^CO_2_ (CO_2_) gas. After injection, the rubber tube and orifice were sealed. In the process of feeding, we turned on the electric fan in the closed marking box in order to ensure that a uniform gas concentration in the box and consistent marking intensity were maintained for oriental melon seedling roots in the same marking box.

We implemented 0.2% (0.2% ± 0.0005%), 0.5% and 1% root-zone ^13^CO_2_ concentration treatments (0.2% is CK, conventional root-zone CO_2_ concentration measured in the early stage; 0.5% and 1% are high root-zone CO_2_ concentrations). The unlabeled 0.2%, 0.5% and 1% CO_2_ concentration treatments were used as the control ^13^C labeled treatments, which were named C-0.2%, C-0.5%, C-1%, ^13^C-0.2%, ^13^C-0.5% and ^13^C-1%, respectively. The sampling of each treatment was repeated three times.

#### 4.2.2. Sampling Period and Method

**Figure 11:**
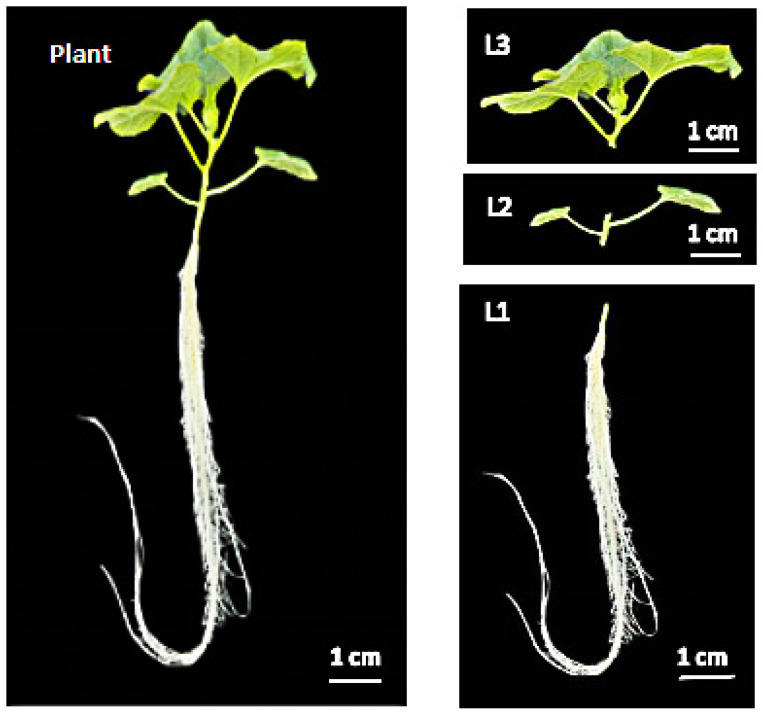
Sampling site of isotopic ^13^CO_2_ feeding plant.

In order to clarify the time limit of CO_2_ absorption by oriental melon roots and transportation to the aboveground part, samples were taken at 0, 0.5, 1.5 and 5 h after feeding. The plants were divided into three parts for sampling (Figure 11). The root was the first part (named L1); the first and second real-leaf and the first and second stems of the plant constituted the second part (named L2), and the other leaves and stems comprised the third part (named L3). Samples were washed in distilled water and dried with filter paper during sampling. The labeled samples were sterilized at 105 °C for 30 minutes, and dried at 75 °C over 72 h. The isotopic composition (δ^13^C) of the sample was measured with an isotope ratio mass spectrometer that was connected to an elemental analyzer (Elementar vario PYRO cube-IsoPrime100, Hanau, Germany).

The results of the ^13^CO_2_ stable isotope tracer experiment showed that oriental melon roots could absorb CO_2_ and transport it to the aboveground part. In order to study the distribution of carbon absorbed by roots in plants under different root-zone CO_2_ concentrations and treatment times, and considering that it was impossible to control the isotope labeling device for a long time, samples were taken at 0, 24 and 72 h after labeling, and the plant was divided into roots, stems and leaves. The treatment method was consistent with the above.

For the determination of carbon assimilation-related enzyme activity and related gene expression, the functional leaves (from the 3rd and 4th nodes above) of oriental melon seedlings that were subjected to 0.2%, 0.5% and 1% treatments were taken at 0, 3, 6, 9, 12 and 15 days after root-zone CO_2_ ventilation treatment. For subsequent analyses, the samples were frozen in liquid nitrogen and stored in a refrigerator at −80 °C.

### 4.3. Measurement Indicators and Methods

#### 4.3.1. Determination of Carbon Content and δ^13^C Value

We weighed and placed the dried samples into the grinding prototype, and ground them through a 100-mesh sieve. Then, we placed 7~8 mg samples into a tin boat and wrapped them, and then determined the carbon content and δ^13^C value of the sample with an EA-IRMS (Elementar vario PYRO cube-IsoPrime100 Isotope Ratio Mass Spectrometer, Germany) (generally speaking, the plant carbon isotope abundance can be expressed by the δ^13^C value). The ^13^C distribution amount, ^13^C distribution proportion and carbon accumulation were calculated using the following formulas:

δ^13^C (‰) = (R_sample_ − R_standard_)/R_standard_ × 1000

where R_sample_ is the ^13^C/^12^C atomic ratio of the sample, and R_standard_ is the ^13^C/^12^C atomic ratio of the standard, which is 0.011802.

^13^C distribution in each organ (mg): ^13^C_1_ = C_1_ (F_i_ − F_il_)/100 × 1000

where C_1_ is the carbon accumulation of each component; F_i_ is the ^13^C abundance of the marker component; F_il_ is the ^13^C abundance of the unmarked component.

Proportion of ^13^C distribution in each organ (%) = ^13^C_1_/^13^C_distribution_ × 100

where ^13^C_distribution_ is the sum of ^13^C distribution of roots, stems and leaves.

Carbon accumulation of each part (mg) = C × 1000 × DW (g)

where C is the carbon content of each part of the root, stem and leaf, and DW is the dry weight of each part.

#### 4.3.2. Determination of Carbon Assimilation-Related Enzyme Activity and Gene Expression

Ribulose-1,5-bisphosphate carboxylase/oxygenase (Rubisco), Rubisco activating enzyme (RCA), fructose-1,6-diphosphate esterase (FBPase), fructose 1,6-bisphosphate aldolase (FBA), sedoheptulose-1,7-bisphosphatase (SBPase) and transketolase (TK) were determined using a kit (Jiangsu Boshen Biotechnology Co., Ltd., Jiangsu, China). Eight genes involved in photosynthetic carbon assimilation were analyzed using qRT-PCR with gene-specific primers. RNA extraction from leaves was carried out according to the instructions for the ultrapure RNA Kit (Beijing Kangwei century biology Co., Ltd., Beijing, China). The synthesis of cDNA was carried out according to the operation method of reverse tran-scription Kit (Monad Biotechnology Co., Ltd., Suzhou, China). Fluorescence quantitative reaction was carried out on a Jena quantitative PCR instrument. The expression amount of each gene was determined by the fluorescence quantitative kit operation method (DRR04A, TANGEN). The PCR reaction procedure used was as follows: 95 °C 30 s; 95 °C 5 s; 60 °C 34 s; 60 °C 15 s, 45 cycles. The relative gene expression was calculated via the 2^−^^△△Ct^ method, the primer sequences are shown in Table 1 and each sample measurement was repeated 3 times.

### 4.4. Statistical Analysis

Data were presented as means ± standard errors (SEs) and analyzed using variance analysis (ANOVA) in SPSS 22.0 (IBM, Armonk, NY, USA). Duncan’s multiple range tests were used to perform significance analysis under conditions of *p* < 0.05. Excel 2010 software was used to perform the data collation and mapping.

## 5. Conclusions

In this study, the isotope tracer test and high root-zone CO_2_ concentration test confirmed that oriental melon roots could absorb CO_2_, and that the root-zone CO_2_ concentration affected plant root carbon absorption and the transportation rate. The carbon absorption and distribution in various organs in oriental melon seedlings were significantly affected by high root-zone CO_2_ concentration. The higher the root-zone CO_2_ concentration, the more carbon was absorbed by the root, the faster the upward transportation speed was, the greater the values in the root, stem and leaf and the higher the proportion that was distributed in the leaf. High root-zone CO_2_ down-regulated the gene expressions of carbon assimilation-related enzymes to affect the activities of carbon assimilation-related enzymes and inhibit the carbon assimilation of oriental melon seedlings.

## Figures and Tables

**Table 1:** Primer list for real-time quantitative PCR.

Gene	Primers Sequences 5′-3′	Accession Number
*Actin*	(F)AAGGCAAACAGGGAGAAGATGA	
	(R)AGCAAGGTCGAGACGTAGGATA	
*CmRubisco*	(F)TCGCAAGAACAACGACATCAC	MELO3C012252.2
	(R)TCACGGTAAACGAATCCACTG	
*CmRCA*	(F)CAACGATGTGGAGGGTTTTTAC	MELO3C008231.2
	(R)TATGTCTGCTGCTTCACGGTAC	
*CmFBA*	(F)AAGGTGCTCGTTTTGCTAAGTG	MELO3C005333.2
	(R)TGTCCTGTCAATGGAATGGTCT	
*CmFBPase*	(F)TCTCGTCGCTTCTCCCTTCA	MELO3C018610.2
	(R)GCCATCACAGCAACTTTTCCA	
*CmSBPase*	(F)GTTCCAGGCTACGAAAGGGT	MELO3C025149.2
	(R)AAATCCCAGATAATCAATGATGCT	
*CmTK*	(F)GGTTCAATCGGGACCGTTTC	MELO3C006200.2
	(R)CCTCAACACCAGGAGTCTCAAAG	
*CmPRK*	(F)ACAGTCTCTACAGCCAAGTCCCT	MELO3C013811.2
	(R)AAGTGCTTTTCCCACACCCT	
*CmPGK*	(F)CTTGGATAGAGCATACCCATACG	MELO3C009351.2
	(R)CAACTCCCCTGGATAACTACACAC	

F: Forward. R: Reverse.
